# Supplementary material for: CryoEM structure of the low-complexity domain of hnRNPA2 and its conversion to pathogenic amyloid
Source: Nat Commun. 2020 Aug 14;11:4090. doi: 10.1038/s41467-020-17905-y (PMC7427792; doi:10.1038/s41467-020-17905-y)
Supplement: Supplementary file 1 — Supplementary Information [file 41467_2020_17905_MOESM1_ESM.pdf]

Supplementary information

CryoEM structure of the low-complexity domain of hnRNPA2 and its conversion to pathogenic amyloid

Jiahui Lu et al.

Jiahui Lu, Qin Cao, Michael P. Hughes, Michael R. Sawaya, David R. Boyer, Duilio Cascio, David S. Eisenberg

Supplementary Figures

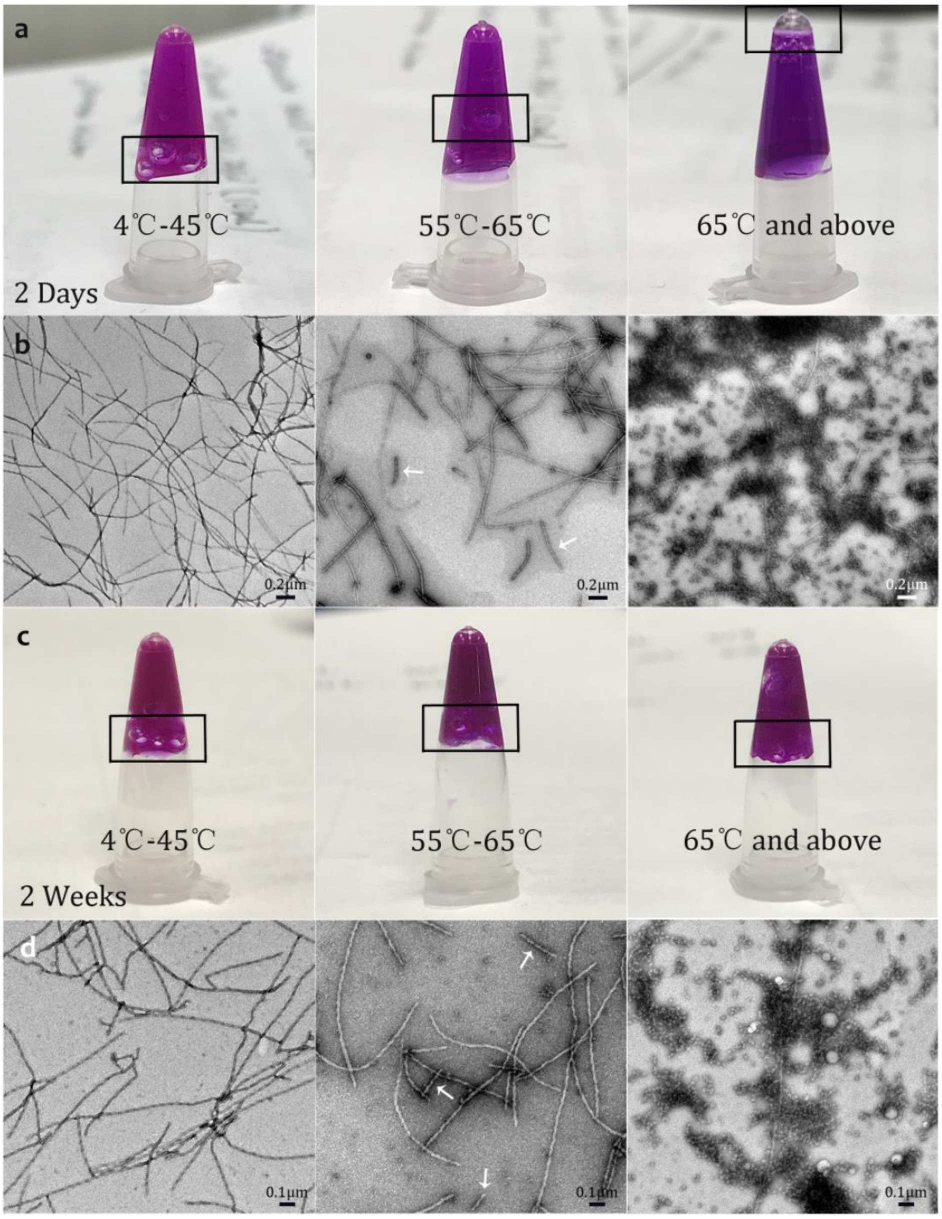

Supplementary Figure 1. Demonstration of lability and reversibility of mC-hnRNPA2-LCD fibrils.

a. A hydrogel formed by concentrated mC-hnRNPA2-LCD stays gel-like while heated from 4 to 45 ℃; it starts to melt at 55 ℃ as shown by movement of the bubble, and becomes a homogenous solution at 65 ℃ and above.

b. Transmission electron micrographs of 2-day hydrogel droplets at three temperatures: 4-45 ℃ hydrogel droplets show uniformly similar, amyloid-like fibrils. 55-65 ℃ hydrogel droplets show fragmented amyloid-like fibrils. 65 ℃ and above droplets show aggregated fibrils and disk-like structures. Scale bars: 0.2 μm

c. The 2-week hydrogel formed by concentrated mC-hnRNPA2-LCD stays gel-like from 4 to 75 ℃ and is thus essentially irreversible.

d. Transmission electron micrographs of the 2-week hydrogel. Scale bars: 0.1 μm

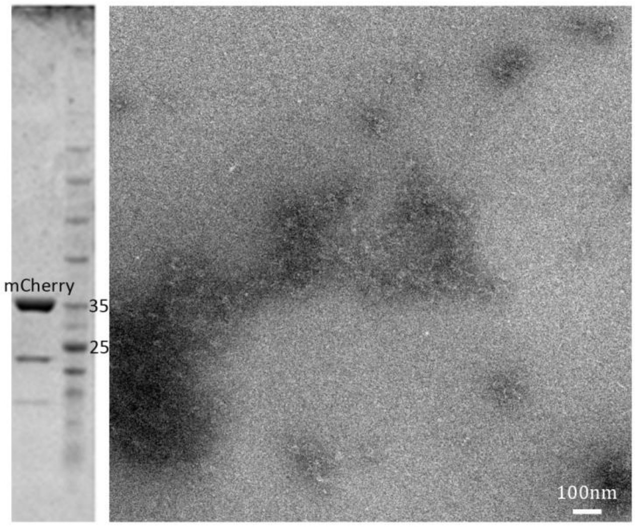

Supplementary Figure 2. Negatively stained TEM image of mCherry.

Left: SDS-PAGE gel with bands showing purified mCherry. Right: Purified mCherry alone heated to 75 ℃ visualized by transmission electron microscopy shows no fibrils or disk-like structures. Scale bars: 100 nm

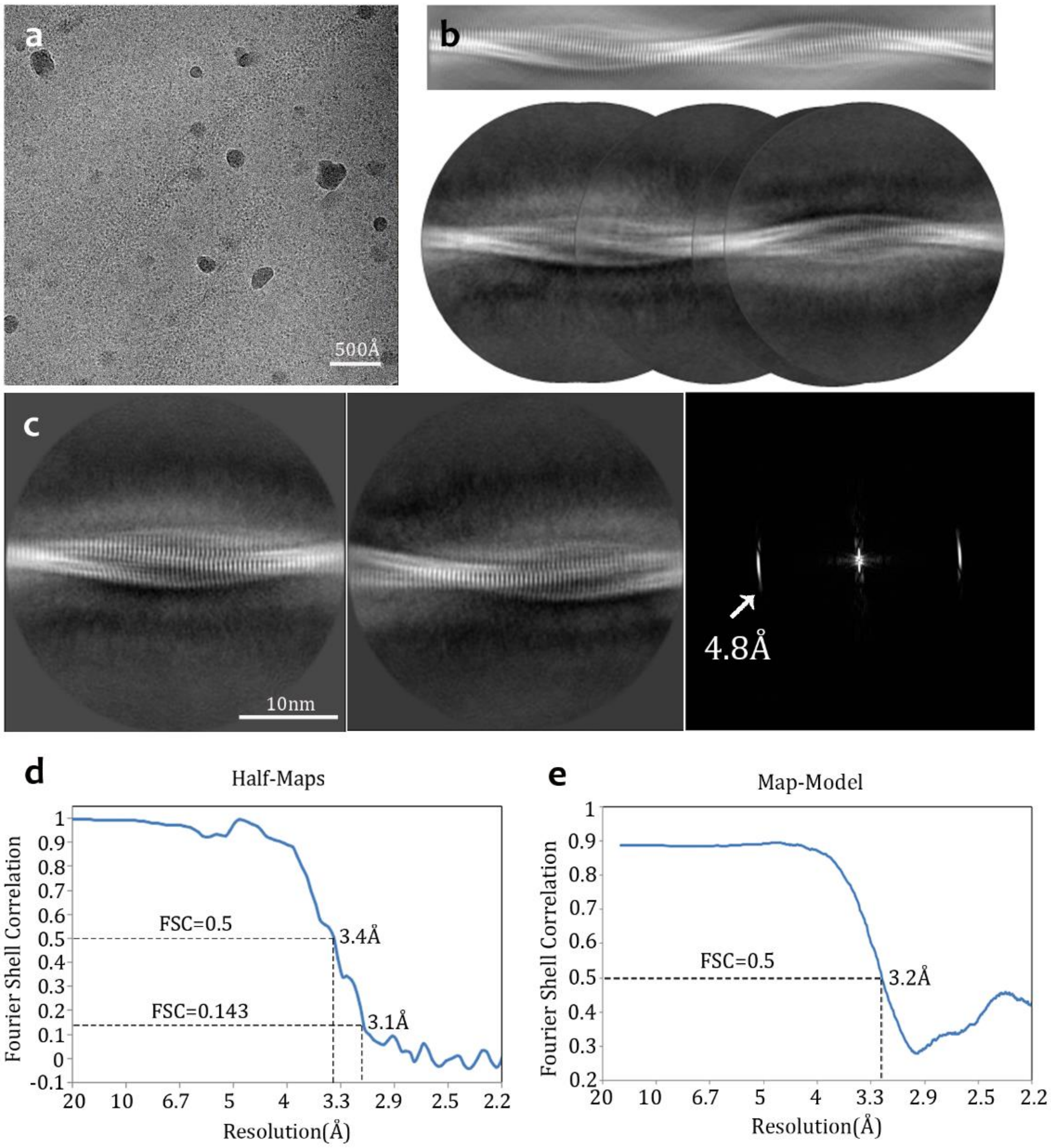

Supplementary Figure 3. Cryo-EM data collection, processing, and refinement of mC-hnRNPA2-LCD

a. One representative micrograph from data collection of mC-hnRNPA2-LCD fibrils. Scale bar: 500 Å

b. Manually assembled full pitch of mC-hnRNPA2-LCD fibril from 2D classification (below) and calculated 2D projection of mC-hnRNPA2-LCD fibril (above).

c. Left and middle: two representative images of 2D classifications showing clear 4.8 Å layers. Scale bar: 10 nm; Right: computed diffraction pattern from 2D class average.

d. FSC curve between two half-maps

e. FSC curve between the cryoEM reconstruction and the refined atomic model

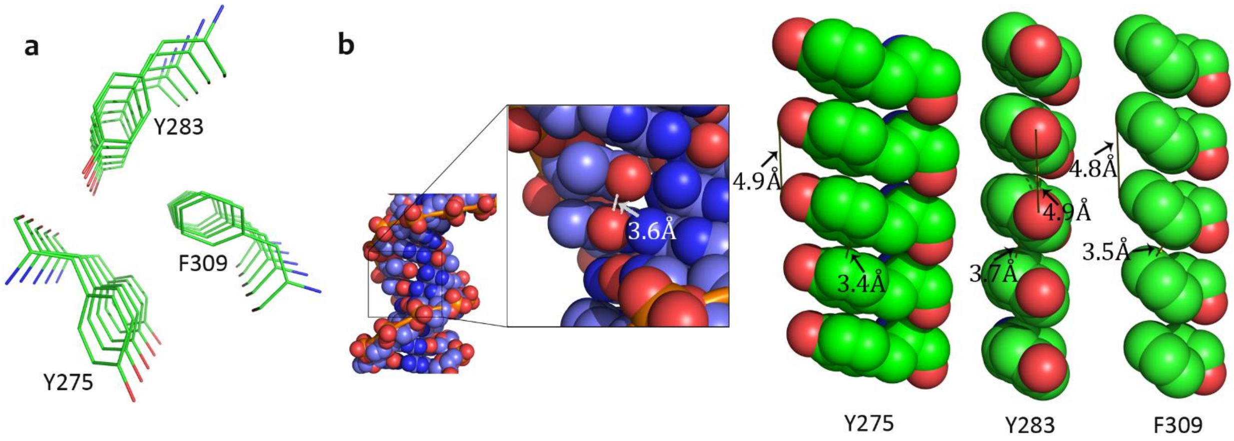

Supplementary Figure 4. Detailed interactions of the aromatic triad

a. View down the fibril axis of five layers of the aromatic triad in mC-hnRNPA2-LCD.

b. Detailed π-stacking interactions shown in Van Der Waals radii for Tyr275, Tyr283, and Phe309 and a control for B-DNA[68]. Y283 and F309 are having partial Van Der Waals radii contact with distances of 3.7 Å and 3.5 Å, respectively.

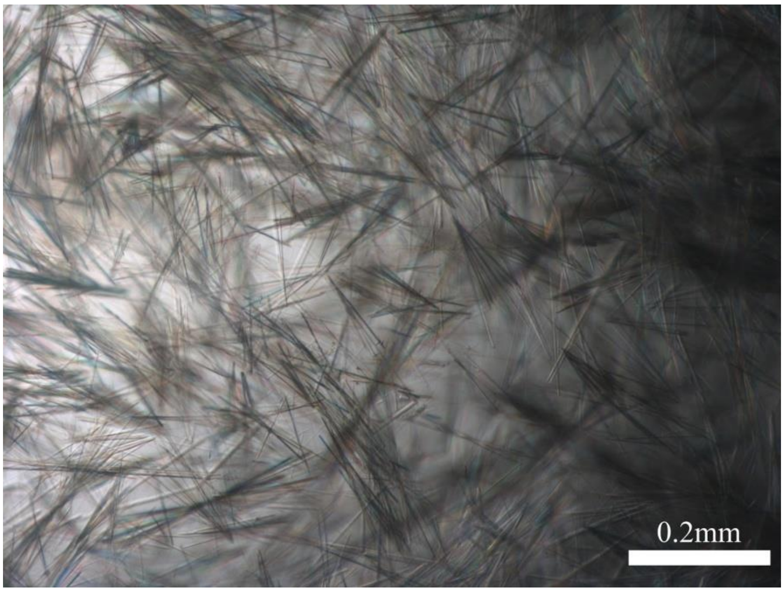

Supplementary Figure 5. GNYNVF mutant segment crystals

Mutant segment GNYNVF needle-like crystals visualized by light microscopy. Crystal growth conditions salt: 0.15 M Ammonium Acetate, precipitant: 35% MPD, buffer: 0.1 M Bis-Tris, pH 5.5. Scale bar: 0.2 mm

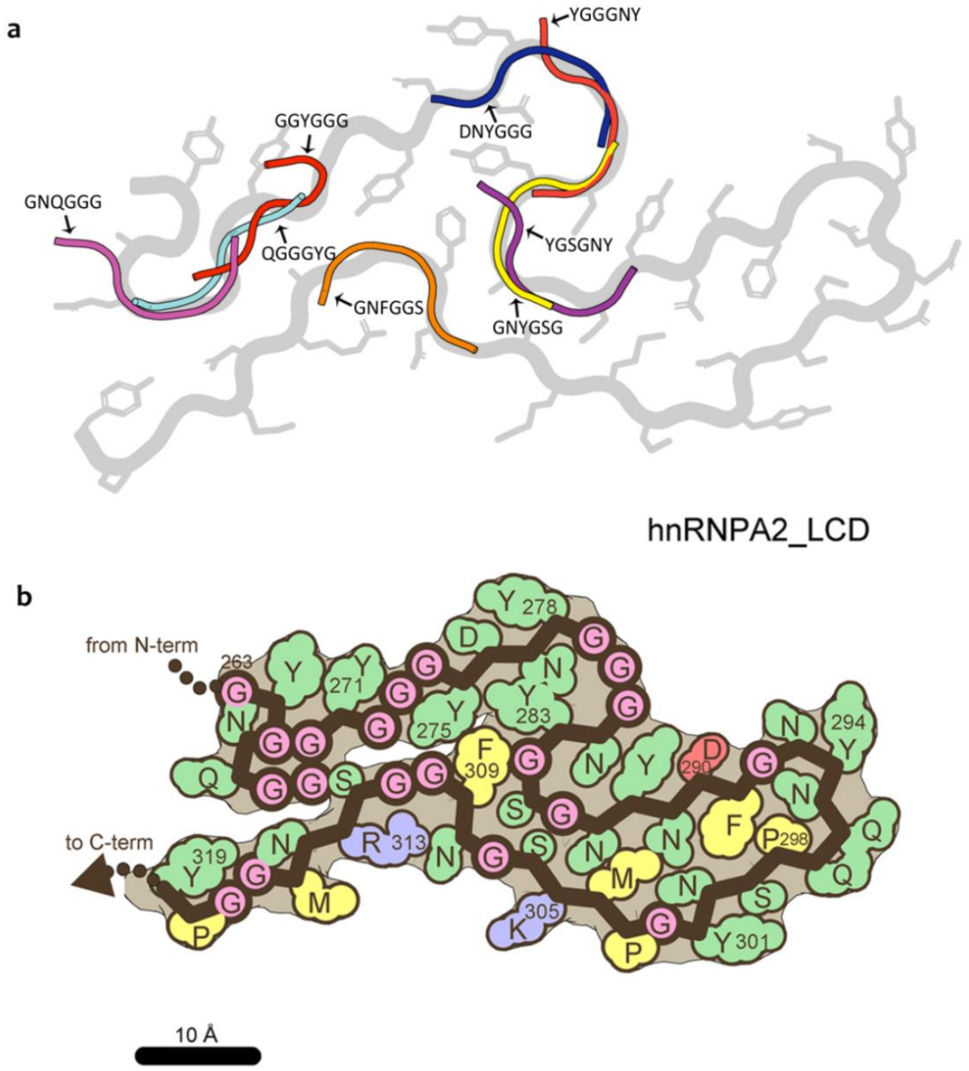

Supplementary Figure 6. Structural analyses reveal the basis of mC-hnRNPA2-LCD's reversibility

a. Superimposition of the backbones of 8 predicted LARKS motifs on the atomic model. Arrows show the calculated LARKS structure backbone and the sequences are indicated. mC-hnRNPA2-LCD structure is shown in gray.

b. Residue polarity map for the atomic model. Hydrophobic residues colored yellow, polar green, glycine pink, glutamate red and arginine blue.

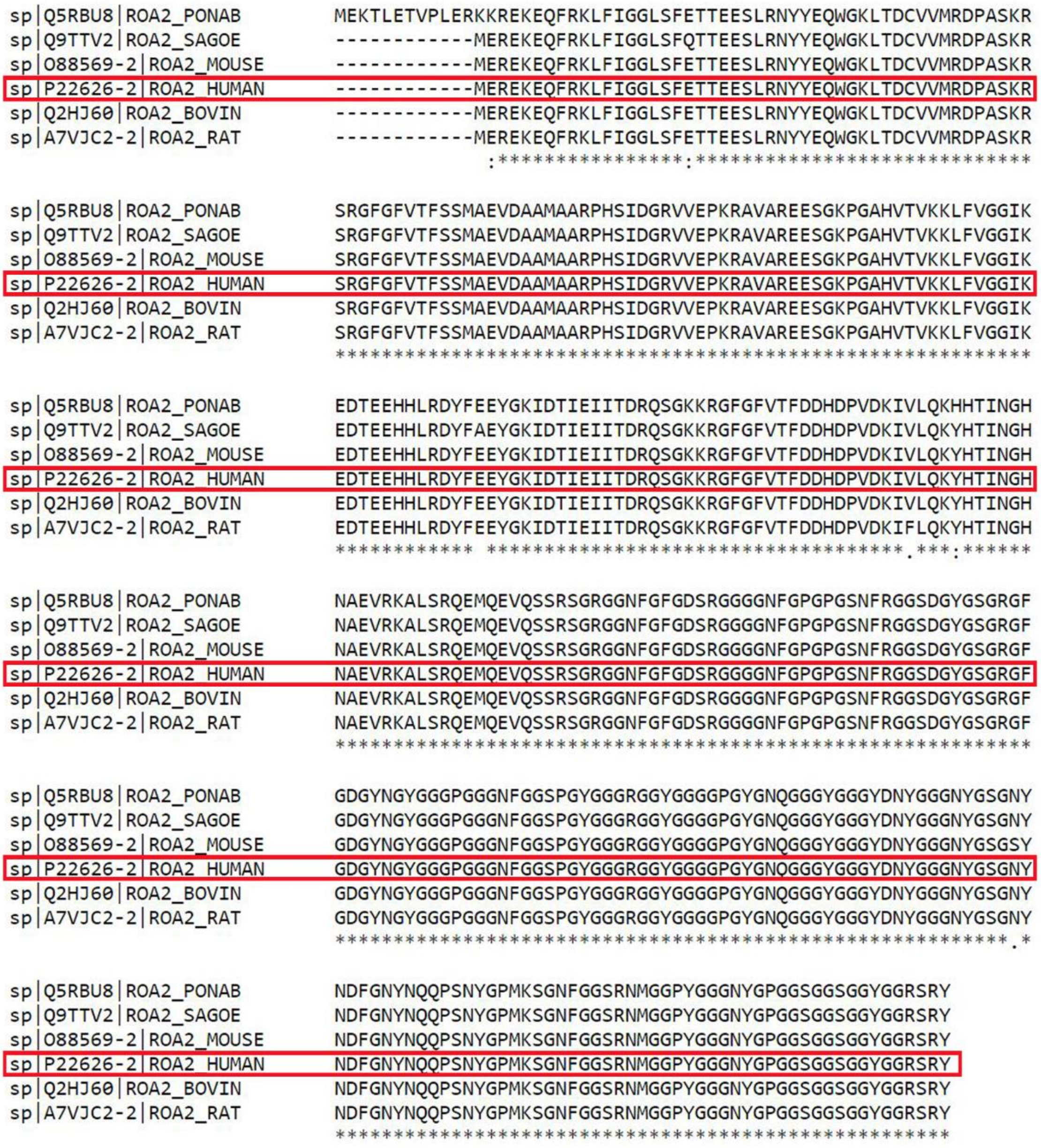

Supplementary Figure 7. Sequence alignment of hnRNPA2 of multiple organisms

Multiple sequence alignment by MUSCLE[69] (https://www.ebi.ac.uk/Tools/msa/muscle/). Human hnRNPA2 sequence is highlighted in red box. PONAB: sumatran oranguta; SAGOE: cotton-top tamarin; BOVIN: bovine. All sequences are from UniProt[70] (https://www.uniprot.org/).

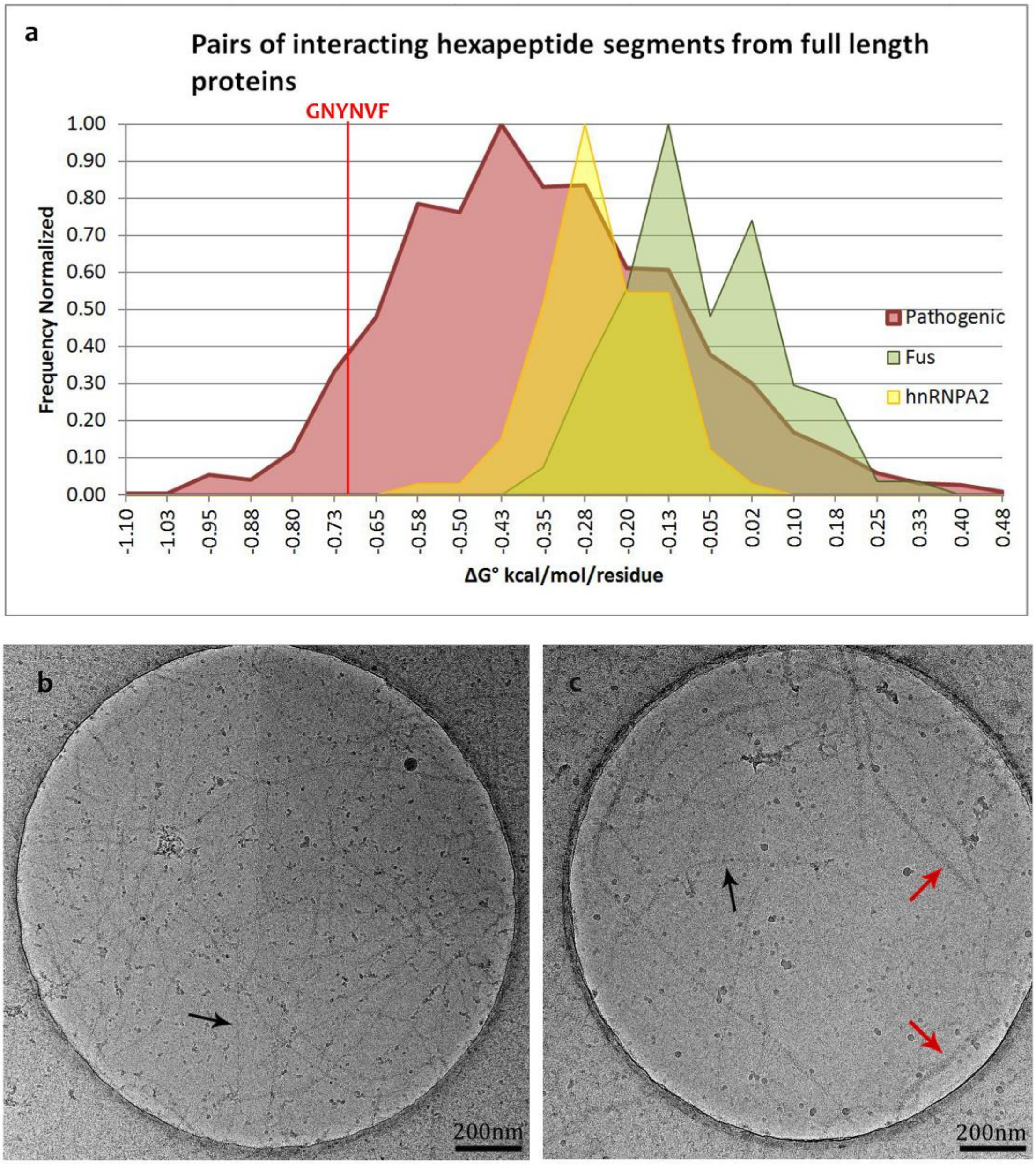

Supplementary Figure 8. Peptide stabilization energy analysis and cryoEM images of wild type and D290V mutant fibrils

a. Histogram of stabilization energies of pairs of interacting hexapeptide segments of hnRNPA2 LCD, FUS LCD, and pathogenic amyloids. The X-axis represents Δ G° in unit of kcal/mol/residue, the Y-axis represents the number of observations. The mutant segment GNYNVF (as a red line) is an outlier in terms of greater stabilization energy compared to reversible proteins.

b. CryoEM image of mC-hnRNPA2-LCD fibrils. Wild type fibrils have homogeneous thickness. Scale bar: 200 nm.

c. CryoEM image of mC-hnRNPA2-D290V-LCD fibrils. Mutant fibrils have a heterogeneous thickness. Black arrow points to fibrils with same thickness as the wild type fibrils. Red arrows point to fibrils with heterogeneous thickness. Scale bar: 200 nm.

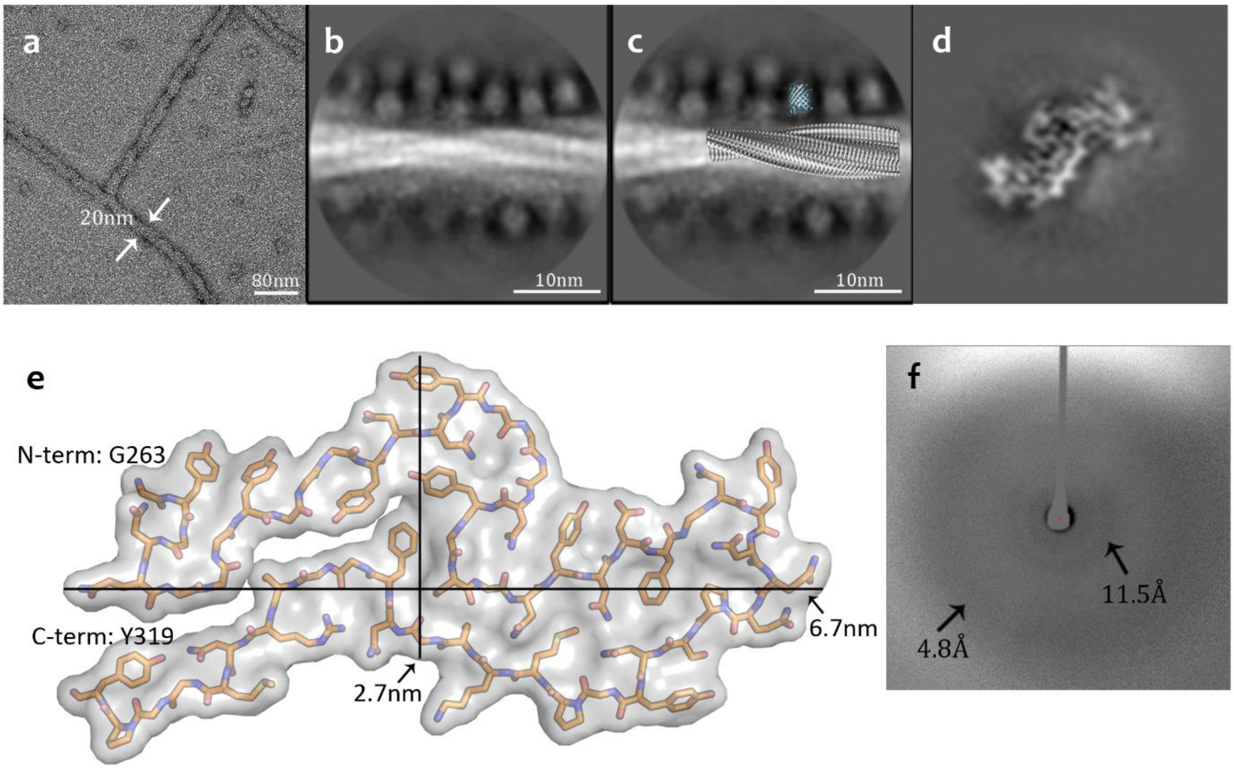

Supplementary Figure 9. mCherry forms a fuzzy coat surrounding the hnRNPA2-LCD fibrils
a. mC-hnRNPA2-LCD fibrils visualized by transmission electron microscopy. Scale bar: 80 nm
b. Representative 2D classification averaged images showing fibrils with a fuzzy coat of spherical blobs.
c. Model built with chimera of the structure of mC-hnRNPA2-LCD (gray) and mCherry (blue) proportional to their real sizes, scaled to B.
d. A central slice from the final 3D reconstruction of mC-hnRNPA2-LCD fibril structure.
e. Surface model of one cross-sectional layer of the fibril, showing that the length of the model is 6.7 nm, and the width is 2.7 nm.
f. X-ray diffraction pattern of mCherry solution (hnRNPA2 not included). Two weak reflections are observed at the same Bragg spacings as the mC-hnRNPA2-LCD hydrogel (Figure 1), but less sharp. That is, the β-barrel architecture of mCherry produces a diffraction pattern with features that overlap with cross-β diffraction. Attention should be paid when doing X-ray diffraction of tagged proteins if performed in the future.

MSYYHHHHHHDYDIPTTENLYFQGAMVSKGEEDNMAIIKEFMRFKVHMEGSVNGHEFEIEGEGEGRPYEGT

QTAKLKVTKGGPLPFAWDILSPQFMYGSKAYVKHPADIPDYLKLSFPEGFKWERVMNFEDGGVVTVTQDSS

LQDGEFIYKVKLRGTNFPSDGPVMQKKTMGWEASSERMYPEDGALKGEIKQRLKLKDGGHYDAEVKTTYKA

KKPVQLPGAYNVNIKLDITSHNEDYTIVEQYERAEGRHSTGGMDELYKAMDPMQEVQSSRSGRGGNFGFGD

SRGGGGNFGPGPGSNFRGGSDGYGSGRGFGDGYNGYGGGPGGGNFGGSPGYGGGRGGYGGGGPGYGNQGGG

YGGGYDNYGGGNYGSGNYNDFGNYNQQPSNYGPMKSGNFGGSRNMGGPYGGGNYGPGGSGGSGGYGGRSRY

Supplementary Figure 10. Protein sequence of mCherry-hnRNPA2-LCD

Protein sequence of mCherry-hnRNPA2-LCD. The magenta-colored sequence corresponds to mCherry, the yellow-colored sequence corresponds to hnRNPA2 LCD.
